# Framework to identify innovative sources of value creation from platform technologies

**DOI:** 10.1073/pnas.2424665122

**Published:** 2025-05-19

**Authors:** Charles H. Jones, Marie Beitelshees, Andrew Hill, Dean Griffiths, Matthew Murphy, Ketan Kapadia, Mikael Dolsten, Jane M. True

**Affiliations:** ^a^Pfizer, mRNA Commercial Strategy & Innovation, New York, NY 10018; ^b^Bulmore Consulting, Lockport, NY 14094; ^c^Clearview Healthcare Partners, New York, NY 10001

**Keywords:** platform technology, drug development, pharmaceutical innovation

## Abstract

Platform technologies are fundamentally reshaping the pharmaceutical industry, offering unprecedented potential for innovation across multiple therapeutic areas. However, traditional valuation models, focused on single-asset metrics, struggle to capture the full spectrum of value these technologies create. This paper presents a comprehensive framework for evaluating the innovative sources of value creation enabled by platform technologies throughout the drug development lifecycle. Through a systematic literature review, in-depth case studies, and framework development, we provide a structured methodology for capturing the diverse benefits of these technologies. Our findings reveal that platform technologies generate value across strategic, technical, and adaptive dimensions, requiring a multifaceted valuation approach. The proposed Platform Value Identification across Strategic, Technical, and Adaptive domains Framework defines key value drivers, specifies quantitative assessment metrics, and provides implementation guidance to inform strategic decision-making in research and development investment, portfolio management, and business development. Application of the framework to case studies of Alnylam’s RNAi platform, Genentech’s therapeutic antibody platform, and Moderna’s mRNA platform demonstrates its broad utility and impact potential. By adopting this holistic, data-driven approach, stakeholders can better assess the long-term value and competitive advantages of well-implemented platform technologies, accelerating the development of transformative therapies for patients.

## Significance

Platform technologies are reshaping drug development by enabling cross-indication adaptability, rapid reuse of data, and faster clinical success. Yet no established framework comprehensively captures the full scope of their strategic and technical value. We address this gap by integrating systematic review, case analysis, and an assessment model to show how platform innovations drive near-term efficiency gains and long-term competitive advantages. Our findings highlight opportunities to quantify intangible value—such as organizational resilience and portfolio diversification—and set a benchmark for future comparative studies. To our knowledge, no existing approach unifies these elements; thus, our framework offers a distinct contribution for stakeholders seeking to optimize platform investments.

The biopharmaceutical industry is undergoing a transformation, driven by platform technologies that are reshaping drug discovery and development. These platforms, spanning RNA therapeutics, precision medicine, and AI-driven discovery, are more than incremental innovations; they represent a rethinking of how pharmaceutical research and development (R&D) is conceptualized and valued. By enabling rapid development of multiple products across therapeutic areas, platform technologies offer efficiencies and strategic advantages in drug discovery, clinical trials, and commercialization (1).

Despite their vast potential, existing valuation frameworks fail to fully capture the multidimensional impact of platform technologies across the pharmaceutical value chain. Traditional valuation methods, which typically rely on linear, single-asset evaluations (2), struggle to recognize the strategic advantages platforms offer, such as the ability to reuse data and processes across various products and the flexibility to adapt to evolving therapeutic needs. Consequently, platform assets often appear undervalued, and their long-term impact is underestimated (3).

The COVID-19 pandemic powerfully demonstrated the transformative potential of platform technologies, with mRNA platforms like Moderna’s enabling the unprecedentedly rapid development of highly effective vaccines (4). This success has further intensified interest in platform technologies, with venture capital investment in platform-based biotech companies nearly doubling from $18 billion in 2019 to $35 billion in 2021 (5). However, realizing the full value of these investments requires a more sophisticated approach to platform technology assessment that captures their unique drivers of competitive advantage.

Through a systematic review of the academic literature on platform technology valuation, in-depth case studies of industry-leading platforms, and development of a valuation framework, we provide a structured methodology for capturing the diverse benefits of these technologies. Our review synthesizes insights from multiple disciplines, spanning biopharma R&D, platform economics and strategy, and technology and innovation management, to identify key value drivers and assessment approaches. The case studies of Alnylam’s RNAi platform, Genentech’s therapeutic antibody platform, and Moderna’s mRNA platform provide rich, real-world examples of how platform technologies create value across strategic, technical, and adaptive dimensions. Building on this evidence base, we propose the Platform Value Identification across Strategic, Technical, and Adaptive domains (VISTA) Framework, which defines core value drivers, specifies quantitative metrics, and offers implementation guidance for holistically assessing platform technologies.

Ultimately, the Platform VISTA Framework provides decision-makers with a critical tool for realigning investment strategies to fully leverage the potential of platform technologies. By offering a structured methodology to evaluate both the tangible and intangible benefits of platforms, this framework enables stakeholders to accelerate innovation, refine financial models, and drive sustained growth across the biopharma industry. While the framework is broadly applicable to diverse enabling technologies, we emphasize its utility for cutting-edge modality-specific platforms like genetic medicines, vaccines, and AI-driven approaches that are redefining the core of drug discovery and development. Our goal is to equip managers and researchers with an actionable approach to guide data-driven decisions and strategies that optimize platform technology value creation and impact.

## Defining “Platform” Technologies

The term “platform technology” encompasses a wide array of tools, systems, and methods used throughout pharmaceutical development (6, 7). For this analysis, we focus on two key categories of platforms that provide distinct advantages across therapeutic areas: molecule-enabled and design-enabled technologies (7). These platform categories are fundamental to modern drug development, driving both biological innovation and operational efficiency (Table 1).

**Table 1.** Comparing platform types

Platform type	Core mechanism	Key features	Applications	Value creation
Molecule-enabled	Biological or chemical modification for therapeutic development	• Modular, adaptable design • Proven MOA • Cross-therapeutic potential	• Genetic disorders • Oncology • Infectious diseases	• Accelerated development through proven biological mechanisms • Reduced risk and cost across multiple indications • Shortened timelines due to modularity and reusability
Design-enabled	Computational, high-throughput, and automated tools for optimizing drug discovery and development	• End-to-end process optimization • Integration across discovery, manufacturing, and clinical trials • Scalability and reusability	• Oncology • Precision medicine • Manufacturing and clinical trial optimization	• Reduced operational costs and timelines • Improved success rates via predictive models • Efficiency gains across R&D and manufacturing

Abbreviations: MOA–Mechanism of Action; R&D–Research and Development.

Molecule-enabled platforms facilitate the development of versatile therapeutics through biological or chemical modifications (7), encompassing modality-specific technologies like RNA therapeutics, antibody-based frameworks, and small molecule platforms. Design-enabled platforms optimize the discovery, development, and manufacturing processes by integrating computational tools, high-throughput systems, and automation (7, 8). Both platform types create value by reducing costs, accelerating timelines, and improving pipeline success rates (Table 1).

While our framework is applicable across these diverse platform technologies, we highlight its particular relevance for cutting-edge modality-specific platforms like genetic medicines (e.g., RNAi, mRNA), therapeutic antibodies, and AI-driven drug discovery. These state-of-the-art platforms are transforming the biopharma R&D landscape and require more nuanced valuation approaches that capture their unique competitive advantages and value creation mechanisms.

## Materials and Methods

### Systematic Literature Review.

To ensure a robust evidence foundation for the VISTA framework, we conducted a systematic literature review following PRISMA guidelines. The review focused on studies related to assessing the value and impact of platform technologies in the biopharma industry.

We searched PubMed, Web of Science, and Scopus using the keywords: (“platform technology” OR “technology platform”) AND (biopharma* OR pharma* OR biotech*) AND (valuation OR assessment OR metrics OR framework). Searches were limited to English language articles published between 2010 to 2024. We also reviewed the bibliographies of key articles to identify additional relevant studies. The initial search yielded 1,524 articles. After removing 552 duplicates, 972 articles were screened by title and abstract against predefined inclusion and exclusion criteria.

Articles were included if they

1) focused on platform technologies in the biopharma industry.
2) addressed approaches to assessing the value or impact of these technologies.
3) provided empirical data, conceptual frameworks, or methodological guidance.

Articles were excluded if they:

1) focused on nonplatform technologies.
2) did not address valuation or assessment.
3) were editorials or commentary without original data or frameworks.

Following screening, 64 articles were reviewed in full. Of these, 38 met all inclusion criteria and were included in the final analysis. Key data were extracted from each article, including the specific platform technology focus, valuation approaches and metrics used, key findings, and implications for best practices in platform assessment. We then analyzed each article for i) platform scope (molecule-enabled vs. design-enabled), ii) primary valuation approaches (quantitative, qualitative, or mixed-method), and iii) key findings relevant to strategic, technical, and adaptive value domains. While multiple references contributed to more than one category of insight, we distilled five recurring themes on platform value creation: 1) multifaceted valuation is essential, 2) standard metrics such as revenue diversification and development efficiency are comparably effective, 3) cross-functional stakeholder engagement strengthens assessment, 4) adaptive value (e.g., ecosystem leadership) is often overlooked, and 5) contextualization within broader industry landscapes refines strategic decisions. Although eight references most directly informed the final articulation of these themes, all 38 studies were integral to developing our holistic perspective. A summary of how each article aligns with these five insights is provided in *SI Appendix*, Table S1.

These findings underscore the importance of a comprehensive, data-driven, and strategically relevant approach to platform valuation, aligning closely with the VISTA framework’s three domains—Strategic, Technical, and Adaptive—and setting the foundation for the subsequent case study analyses.

The systematic review revealed several key insights that inform the VISTA framework:

1. Platform technologies generate value across strategic, technical, and adaptive dimensions, requiring a multifaceted approach to valuation (9, 10).
2. Quantitative metrics related to factors such as revenue diversification, development efficiency, and intellectual property strength are critical for comparing platform value and impact (11).
3. Successful platform assessment requires a structured and iterative process that engages cross-functional stakeholders and leverages diverse data sources (12, 13).
4. Adaptive value is an oft-overlooked but essential aspect of platform competitive advantage, reflecting dynamic capabilities and ecosystem leadership (14, 15).
5. Platform valuations must be contextualized within the broader biopharma market and technology landscape to inform actionable strategic insights (16).

These findings underscore the importance of a comprehensive, data-driven, and strategically relevant approach to platform valuation, aligning closely with the VISTA framework domains and metrics. The systematic review supports the face validity and applicability of the framework while providing key inputs to refine its components and implementation guidance.

## How Can Platform Technologies Drive Value?

**Fig. 1.**
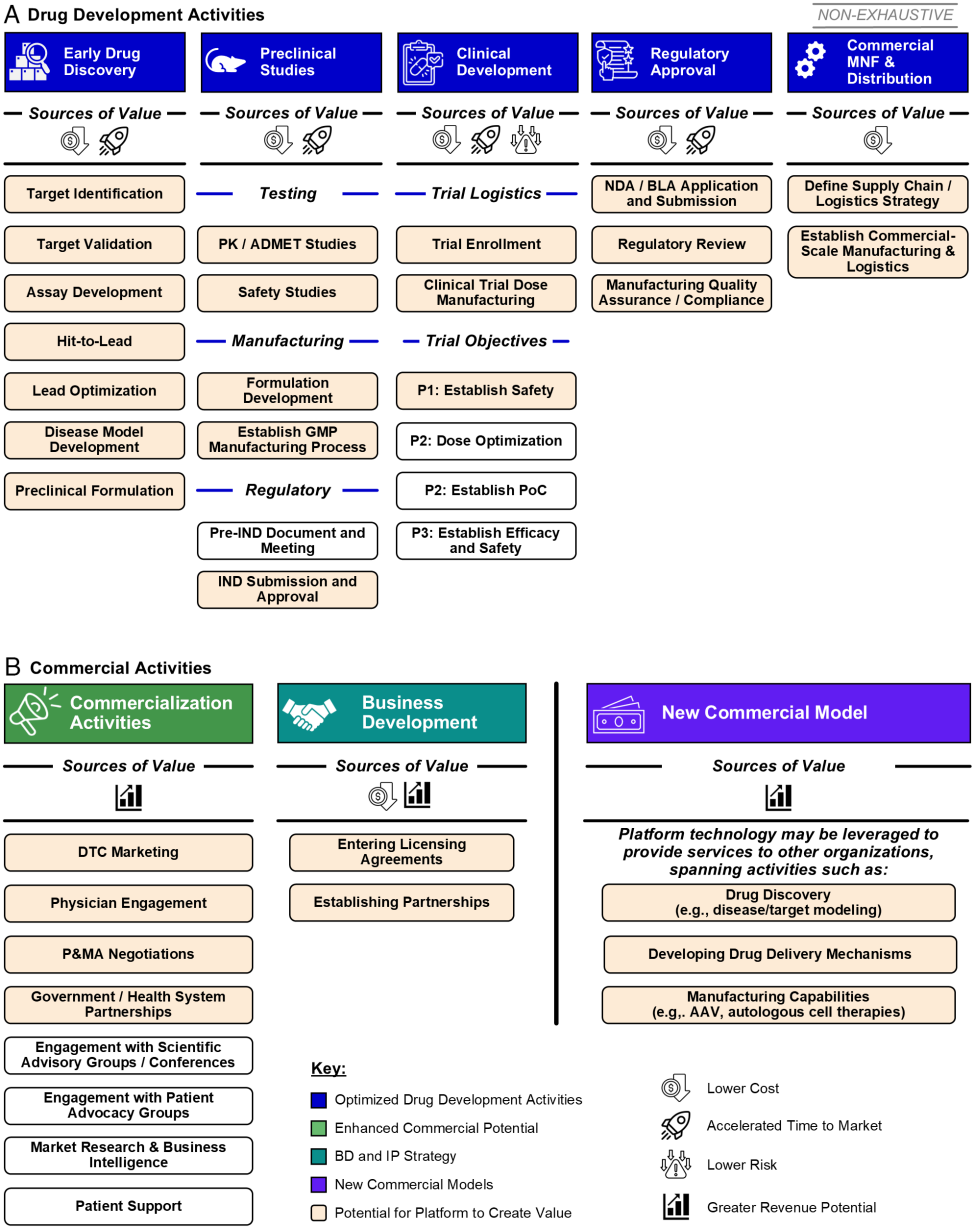
Where platform technologies drive value across the pharmaceutical value chain. (*A*) Drug development activities that drive value across all phases of drug development from early drug discovery to manufacturing. (*B*) Commercialization and business development activities that drive value and new commercial models facilitated by platform technologies.

Platform technologies are revolutionizing pharmaceutical development by introducing modular, scalable, and reusable frameworks that transcend traditional single-asset approaches. Here, we explore both the tangible and intangible value generated by platform technologies throughout the drug development lifecycle (Fig. 1) and highlight how these innovations potentially offer superior returns over traditional models. These insights set the stage for a comprehensive framework to capture the immediate and strategic value created by platform-based approaches.

### Incremental Value Creation in Drug Development.

The traditional drug development process is lengthy, costly, and high-risk, often taking over a decade and requiring more than $1 billion to bring a single drug to market (17). Platform technologies introduce a modular and scalable approach that is revolutionizing this process by reusing data, validated mechanisms of action, and implemented automation to accelerate multiple candidates simultaneously, delivering significant value from early discovery to clinical development (7, 9).

#### Accelerating early drug discovery.

One of the most transformative aspects of platform technologies is their ability to accelerate early drug discovery through design-enabled capabilities. Traditional early-stage discovery involves labor-intensive screening of compound libraries to identify and optimize potential therapeutic leads, a process that typically takes years with a high probability of failure at each stage (17). In contrast, design-enabled platforms leverage high-throughput screening (HTS) and AI-driven predictive models to streamline this process (18, 19). These platforms can screen hundreds of thousands of compounds daily, allowing researchers to identify promising lead candidates much more rapidly. For example, platforms utilizing genetic sequence-based targeting have demonstrated a 3 to 8 mo advantage in time to clinical assets and a 30% reduction in R&D costs compared to traditional approaches, resulting in two to three additional clinical candidates every 5 y (1).

AI-driven drug design further enhances the efficiency of early drug discovery by predicting molecular interactions with high precision, thereby reducing the trial-and-error inherent in traditional approaches (19, 20). Advanced AI models embedded in platforms can process multidimensional datasets, continuously refine predictions, and improve the identification of lead candidates. A notable example is Insilico Medicine, which used its AI-driven platform to discover a novel drug candidate for idiopathic pulmonary fibrosis in just 18 mo—a stark contrast to the conventional 4-to-5-y timeline—at only 10% of the cost (21). Similarly, Exscientia, in collaboration with Sumitomo Pharma, employed its AI-based platform to identify a new molecule targeting obsessive-compulsive disorder (OCD) and advanced it to Phase 1 clinical trials in just 12 mo, significantly faster than traditional drug discovery methods (22).

The 2024 Nobel Prize in Chemistry, awarded for the application of AI to protein structure prediction and protein–ligand complex modeling, underscores the transformative potential of AI in accelerating drug discovery (23). These advances are expected to accelerate drug design across multiple dimensions, including reduced timelines, lower costs, and enhanced ability to address complex therapeutic targets.

By enabling the reuse of datasets, integrating HTS, and deploying advanced AI models, platform technologies drastically reduce the time and costs associated with early drug discovery while simultaneously increasing the accuracy and success rates of identifying lead candidates. This modularity and scalability streamline early-stage development and set the foundation for more efficient progression through subsequent phases, providing significant incremental value throughout the drug development lifecycle.

#### Reducing redundancies in preclinical and IND-enabling activities.

Preclinical development is often a time-consuming phase of drug development, as it requires extensive safety and pharmacokinetic (PK) testing to ensure that a drug candidate is suitable for human trials (17). Traditionally, each drug candidate undergoes repetitive and labor-intensive testing, resulting in significant bottlenecks. Molecule-enabled platforms, however, offer a more efficient path by allowing companies to reuse validated MOAs and delivery systems, thereby streamlining the route to Investigational New Drug (IND) submissions (6).

The U.S. FDA issued draft guidance titled “Platform Technology Designation Program for Drug Development” in May 2024 (24). This guidance establishes a framework for employing platform technologies in drug development, specifically permitting the reuse of nonclinical safety data within a platform program. Once a platform has established a validated safety profile, these data can be leveraged for other candidates developed within the same platform, drastically reducing the need for repetitive preclinical tests. This efficiency is particularly evident in technologies like mRNA platforms, where safety data demonstrated in one therapeutic area can be applied to others (25). As a result, preclinical timelines can be shortened by approximately 25%.

By enabling the reuse of validated mechanisms of action and benefiting from regulatory support, molecule-enabled platforms effectively reduce redundancies in preclinical and IND-enabling activities. This saves time and resources while maintaining high safety standards, ensuring drug candidates can advance through the development pipeline more efficiently. As a result, platform technologies significantly enhance the overall value and efficiency of the drug development process.

#### Accelerating clinical development.

Clinical trials represent the most resource-intensive and time-consuming phase of drug development, often associated with high failure rates and lengthy patient recruitment processes (26). Platform technologies, however, provide a significant advantage in accelerating clinical development by leveraging validated MOAs and established safety profiles. This reliance on preexisting data not only reduces trial risks but also expedites patient recruitment, resulting in faster development timelines and more efficient progression through clinical phases.

One of the most impactful improvements offered by platform technologies is the increased success rate in clinical trials. Traditional single-asset trials face high levels of uncertainty, particularly in early phases, which contribute to a low overall success rate. In contrast, platform-based approaches build upon validated safety and efficacy data, enhancing the probability of success for subsequent candidates. Moderna’s mRNA platform is a prime example of this benefit, having achieved a 70% success rate in Phase 1 trials compared to the industry standard of 35% (27). This remarkable increase is attributable to the platform’s ability to reuse established safety profiles, thereby minimizing the uncertainties that typically hinder early-phase trials and ensuring a more streamlined path toward clinical development.

Another critical advantage of platform technologies is their ability to accelerate patient recruitment. Patient recruitment is often a major bottleneck, as trials can face significant delays due to the time required to identify and enroll eligible participants. Platforms that have gained trust within the healthcare ecosystem, such as Alnylam’s siRNA platform, have demonstrated the capacity to expedite this process. According to clinical trial data, Alnylam’s platform reduced recruitment times by approximately 2 mo for Phase 1, 5 mo for Phase 2, and 17 mo for Phase 3 (10K Statements), likely driven by clinician confidence in the platform’s established safety data. This trust enables more rapid enrollment and fosters a smoother recruitment process, which in turn accelerates the overall clinical development timeline.

By leveraging validated safety profiles and building trust among clinicians and patients, platform technologies reduce trial failure rates and expedite patient recruitment. This leads to faster clinical development and earlier market entry, significantly enhancing the efficiency and success of drug development. Platform-based approaches thus provide a strategic advantage by streamlining the most resource-intensive phase of the drug development process, ultimately driving value and accelerating the journey from clinical trials to market approval.

#### Streamlining manufacturing.

Manufacturing in the pharmaceutical industry is traditionally a complex and resource-intensive process, requiring dedicated equipment, infrastructure, and protocols for each new drug (28). This approach often leads to increased costs, longer timelines, and inefficiencies, as every asset demands a unique manufacturing process. Platform technologies offer a transformative solution by enabling shared manufacturing processes, significantly reducing time, costs, and complexities across the drug development value chain.

The primary improvement offered by platform technologies in manufacturing is the efficiency gained across multiple product lines. By utilizing shared infrastructure and processes, manufacturers can leverage existing equipment and facilities for various products developed within the same platform, eliminating the need for developing entirely new manufacturing lines for each drug (28). This stream lining allows for a faster and more seamless transition from preclinical formulation to clinical-scale production and eventually to commercial manufacturing.

Platform technologies also facilitate accelerated production timelines, a critical advantage in responding to market demands or emerging health crises. For example, Pfizer’s mRNA platform demonstrated the ability to adapt to the changing landscape of the COVID-19 pandemic. The platform enabled the rapid modification of its vaccine to address new viral strains, compressing the process from strain modification to large-scale production into just 100 d (28). This adaptability is made possible by the standardized protocols inherent in platform technologies, allowing for quick adjustments and scaling without requiring extensive revalidation or process overhaul.

In addition to accelerating production, platform technologies can lead to significant cost reductions. By standardizing manufacturing processes and minimizing the need for customized approaches, platforms optimize supply chain logistics and reduce the overall cost of goods sold (COGS). The uniformity across products also simplifies quality control and regulatory compliance, further driving down costs and improving efficiency.

By enabling the use of shared manufacturing infrastructure, standardized protocols, and established processes, platform technologies enhance manufacturing efficiency, allow for quicker market entry, and enable rapid adaptation to changing needs. This scalability and flexibility create substantial value across the manufacturing phase of drug development, making platform technologies a critical driver of streamlined, cost-effective, and responsive pharmaceutical production.

### Incremental Value Creation in Commercial Operations.

Beyond accelerating the drug development process, platform technologies offer significant commercial advantages by opening up new avenues for revenue generation and strategic expansion. Unlike traditional single-asset models, platforms provide unparalleled scalability and flexibility, allowing companies to diversify their revenue streams through outlicensing, strategic partnerships, and the expansion of their product portfolios. Companies with platform technologies can also benefit from an “innovation premium,” or the additional value that investors are willing to pay for a company due to its perceived potential for future innovation and growth (29).

One of the most tangible ways platform technologies create commercial value is through licensing and strategic partnerships. The scalability inherent in platform technologies enables companies to license their platforms to external partners, generating substantial revenue while simultaneously advancing their internal pipeline. Arrowhead Pharmaceuticals, for example, has capitalized on its innovative platform technologies, such as the Targeted RNAi Molecule (TRiM™) platform for RNAi therapeutics, to forge several profitable partnerships and outlicensing agreements, including with Amgen, Takeda, Horizon, and GlaxoSmithKline Plc (GSK) (30–33). By leveraging this model, Arrowhead Pharmaceuticals not only mitigated internal development risks but also broadened its market reach, demonstrating how platform technologies can serve as a powerful engine for commercial growth. This approach allows companies to benefit from external funding and expertise, accelerating the development and commercialization of their platform-based products while maintaining a strong internal focus on innovation.

Platform technologies also facilitate faster market penetration, a crucial factor for commercial success, stemming from the familiarity and trust that healthcare professionals and patients develop with the platform over time (34). This established trust makes healthcare providers more inclined to adopt and recommend new therapies associated with a recognized platform, even when those therapies fall outside their primary area of expertise. As each new offering based on a validated platform enters the market, its linkage to a well-established technology can result in faster adoption rates compared to standalone assets that often face skepticism and delays.

By offering scalability and adaptability, platform technologies allow companies to develop multiple candidates, generate revenue through external partnerships, and penetrate markets more efficiently. This capability to diversify revenue streams and facilitate faster market entry highlights the immense commercial value that platforms bring, making them a vital component for long-term growth and success in the pharmaceutical industry. As such, platform technologies not only transform the development process but also redefine how companies can capitalize on their innovations in the marketplace.

### Intangible Value of Platform Technologies.

In addition to the tangible operational and commercial benefits, platform technologies offer substantial intangible value that contributes to long-term competitive advantages for pharmaceutical companies. These intangible assets enhance a company’s market position and create a sustainable foundation for future growth by attracting top-tier talent, fostering continuous innovation, and building robust intellectual property (IP) portfolios that act as barriers to entry for potential competitors.

One of the most significant ways platform technologies create intangible value is by attracting talent. Companies that pioneer platform technologies are often viewed as innovation leaders within the industry, making them highly attractive to top scientists and researchers who seek opportunities to work on cutting-edge projects (35). Moderna’s leadership in mRNA technology has exemplified this effect, establishing a once-unknown company as a desirable biotech employer and drawing top-tier talent to its ranks (35). This influx of highly skilled personnel enhances the company’s innovation pipeline and fosters a self-reinforcing cycle of discovery, where the expertise and creativity of new recruits contribute to continuous advancements in the platform, further solidifying the company’s position as an industry leader.

In addition to attracting talent, platform technologies play a critical role in building extensive IP portfolios that serve as formidable barriers to competition. A strong IP portfolio not only protects a company’s proprietary technologies but also secures its market leadership by preventing competitors from developing similar therapies without entering costly licensing agreements. Alnylam’s RNAi platform, for example, is protected by a broad portfolio of 2,500+ granted patents worldwide, making it difficult for competitors to replicate or develop similar RNAi-based therapies (36, 37). This extensive IP coverage ensures that Alnylam retains exclusive rights to its technology, enabling the company to maintain its competitive advantage and capitalize on the value generated by its platform over time.

**Fig. 2.**
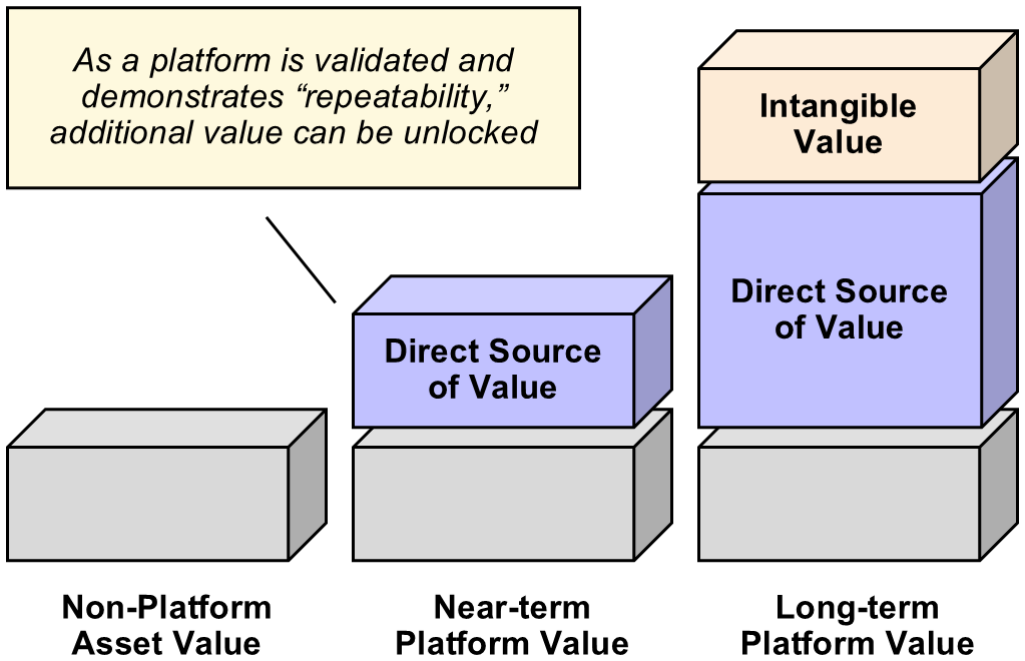
Pharma technology value creation. Validated and repeatable approaches in platform technologies can unlock additional value. Direct sources of value expand with consistent validation, and intangible value builds upon this foundation, driving greater long-term impact.

The ability of platform technologies to foster innovation and protect IP stems from their inherent reusability and scalability (Fig. 2). As platforms enable the development of multiple drug candidates across different therapeutic areas, they continually expand a company’s IP assets and enhance its ability to innovate. This positions platform-based companies as market leaders, allowing them to secure long-term market dominance and create significant intangible value that extends beyond immediate commercial returns. By nurturing talent, driving innovation, and building a robust IP portfolio, platform technologies provide pharmaceutical companies with the strategic advantages needed to thrive in a competitive landscape, ensuring sustained growth and leadership in the industry.

### A Framework for Measuring “Value” (Valuation) of Platform Technologies.

Platform technologies are transforming pharmaceutical R&D by introducing scalable, modular approaches that overcome traditional drug development constraints. These technologies offer a multidimensional value that conventional valuation models often underestimate. To bridge this gap, we propose a comprehensive Platform VISTA Framework (Table 2 and *SI Appendix*, Table S2) that enables a systematic, nuanced evaluation of the value created through the use of platform technologies, differentiating between molecule-enabled and design-enabled platforms. This framework provides a structured approach to capturing the value these platforms bring throughout drug discovery, clinical development, manufacturing, and commercialization, supporting more informed decision-making and strategic investment.

**Table 2.** The platform VISTA framework

Area of value creation	Molecule-enabled platforms	Design-enabled platforms	Key metrics of value creation
I. Drug development			
Target & lead discovery	Validated MOAs, cross-indication adaptability	AI-driven screening & predictive modeling; HTS	Lead ID time, viability rate, efficacy, cost-efficiency
Lead optimization	Modular design, cross-therapeutic adaptability	Computational prediction accuracy; enhanced HTS precision	Development time, optimization speed, efficacy improvement
Clinical trial design	Reusable safety profiles; reliable mechanisms	Data-driven design; optimized patient selection	Trial success, recruitment & retention, cost reduction
Accelerated development	Reduced early-phase redundancy	IND-enabling data integration; safety/efficacy prediction	IND speed, regulatory success, time-to-trial initiation
Process efficiency	Standardized scalable production	Automated & digital quality control; consistent protocols	Cost/batch, production speed, process adaptability
Scalability & flexibility	Shared infrastructure; modular scalability	Workflow automation; digital process control	Scale-up, batch consistency, cost-savings, adaptability
II. Commercial value			
Revenue generation	Flexible product portfolio; outlicensing; strategic partnerships	Licensing-based revenue; market expansion	Revenue diversity, partnership revenue, market penetration rate
Market entry	Speed to market; cross-use across indications	Licensing-based expansion; market adoption due to validated efficacy	Launch time, market penetration speed, geographic reach
IP portfolio	Broad patent coverage; platform exclusivity	Algorithm/process patents; Data privacy and IP protection	IP strength index, FTO, competitive IP position
Innovation platform	Deepening R&D expertise; Diversification in therapeutic areas	Advanced computational tools; algorithmic optimization; platform evolution	Innovation rate, tech advancement, cross-therapeutic adaptability
III. Intangible value			
Talent acquisition & retention	Innovation reputation; domain expertise	Attraction of top-tier AI/ML and data scientist talent	Talent retention, recruitment, innovation productivity
Knowledge expansion	Insights into biology, clinical & manufacturing	Cross-project data sharing; algorithm refinement	Knowledge sharing, cross-learning, transfer efficiency
Industry leadership	Pioneering MOAs; reputation for clinical reliability	Recognized tech leadership & operational efficiency	Partnerships, brand influence, collaboration demand
Platform evolution	Indication expansion; adaptability	Improved model precision & platform capabilities	Platform enhancement rate, new use adaptability, appreciation in platform value

Abbreviations: AI; AI/ML—AI/Machine Learning; FTO—Freedom to Operate; HTS—High-Throughput Screening; IND—Investigational New Drug; IP—Intellectual Property; MOA—Mechanism of Action; R&D—Research and Development.

The VISTA framework consists of three core value domains that collectively capture the multifaceted impact of platform technologies: Strategic, Technical, and Adaptive. The strategic domain encompasses the value generated through business model innovation, revenue diversification, and ecosystem leadership enabled by platform technologies. The technical domain focuses on the value created through enhanced product performance, accelerated development timelines, and streamlined manufacturing processes. Finally, the adaptive domain captures the value of organizational capabilities and culture that support continuous learning, agility, and resilience in dynamic market contexts.

To operationalize the assessment of value across these domains, the VISTA framework specifies quantitative metrics and a structured implementation approach. Each domain includes three to five core metrics that can be calculated using standard industry data to enable benchmarking and comparability. The framework also provides step-by-step guidance on data collection, metric calculation, and triangulation across domains to facilitate a robust, holistic evaluation. The following sections detail the value domains, metrics, and implementation process with illustrative examples.

### Strategic Value Domain.

The strategic value domain captures the business model and ecosystem-level implications of platform technologies. It includes value generated through factors such as expanded market access, diversified revenue streams, and industry leadership. Core metrics for assessing strategic value include:

- Revenue Diversification Index (RDI): Measures the distribution of revenue across therapeutic areas, geographies, and business models, calculated as RDI = 1 − ∑(Segment Revenue/Total Revenue)^2^.
- Partnership Strength Index (PSI): Assesses the quality and impact of platform partnerships, calculated as PSI = ∑ [(Partner Tier Score × Partnership Type Score × Deal Value)/# of Partnerships].
- IP Leadership Score (IPLS): Evaluates the strength and scope of the platform’s intellectual property portfolio, calculated as IPLS = (Patent Citation Frequency × Jurisdictional Coverage × Technological Scope)/1,000.

To illustrate, consider the case of Alnylam’s RNAi therapeutic platform. As of 2022, Alnylam had an RDI of 0.75, indicating strong diversification across four therapeutic areas and three major geographies. Its PSI was 38.4, reflecting a robust network of 10+ partnerships with top-tier pharma companies across codevelopment, licensing, and research collaboration deals. Alnylam’s IPLS of 7.2 captures its industry-leading RNAi patent portfolio, with 2,500+ granted patents worldwide cited at 1.5× the industry average rate. Together, these metrics underscore the significant strategic value of Alnylam’s platform.

### Technical Value Domain.

The technical value domain assesses the operational efficiencies and performance improvements enabled by platform technologies across the drug development lifecycle. Core metrics include:

- Probability of Technical Success (PTS): Measures the likelihood of a drug candidate progressing from preclinical development through each clinical trial phase to regulatory approval, relative to industry benchmarks.
- Cycle Time Efficiency (CTE): Evaluates R&D productivity in terms of the speed and cost of moving drug candidates through each development stage, calculated as CTE = Industry Benchmark Cycle Time/Actual Cycle Time.
- Manufacturing Efficiency Ratio (MER): Compares the platform’s manufacturing cost and scale performance to industry standards, calculated as MER = [1 − (Actual COGS/Industry Benchmark COGS)] × [Actual Batch Size/Industry Benchmark Batch Size].

For example, an analysis of Genentech’s therapeutic antibody platform shows a PTS 1.3× higher than industry averages, with 63% of preclinical candidates reaching Phase 3 compared to 48% for nonplatform antibodies. The platform’s CTE is 1.6, indicating 60% faster development speed, with an average of 6.2 y from discovery to approval vs. 10+ y typical in biopharma. Genentech’s MER of 3.5 reflects its ability to manufacture antibodies at 70% lower COGS and 5× the scale of industry benchmarks, leveraging platform standardization and efficiencies.

### Adaptive Value Domain.

The adaptive value domain captures the dynamic capabilities and organizational learning enabled by platform technologies that support long-term competitive advantage. Key metrics include:

- Innovation Velocity Index (IVI): Assesses the rate and impact of new product launches and indication expansions, calculated as IVI = (# of New Launches × Average Peak Sales Potential)/Years Since Platform Inception.
- Talent Leverage Ratio (TLR): Measures the firm’s ability to attract, develop, and retain top talent to sustain platform leadership, calculated as TLR = (Employee Engagement Score + Innovation Culture Score)/Industry Benchmark.
- Ecosystem Evolution Score (EES): Evaluates the platform’s ability to create value through generative interactions with external stakeholders, calculated as EES = Partner Renewal Rate × Partner Referral Rate × Ecosystem Satisfaction Score.

Moderna’s mRNA platform exemplifies adaptive value, with an IVI of $7.5B reflecting ten clinical programs progressing in parallel with an average peak sales potential of $1.5B, just 3 y after platform validation. Moderna’s TLR of 2.3 captures its top-decile employee engagement and innovation culture scores, supported by a highly skilled, entrepreneurial 1,800-person organization. Its EES of 4.2 reflects a 90% partner renewal rate, 50% new deals from referrals, and industry-leading satisfaction scores, highlighting the platform’s ability to coevolve with its ecosystem.

### Implementing the VISTA Framework.

To apply the VISTA framework, organizations should follow a four-step process:

1. Define the scope and strategic objectives of the platform technology assessment.
2. Collect data on the relevant metrics for each value domain using internal and external sources.
3. Calculate the metrics and compile them into a balanced scorecard, identifying areas of strength and opportunity.
4. Triangulate insights across the value domains to develop an overall assessment and recommendations for optimization.

To support effective implementation, organizations should establish a cross-functional VISTA assessment team with representation from R&D, commercial, manufacturing, and business development. The team should align on the goals and timeline for the assessment, and establish processes for data governance, updates, and decision-making. On an ongoing basis, the team should revisit the assessment to track progress and identify new value creation opportunities.

By integrating quantitative metrics and a structured approach to assessing strategic, technical, and adaptive value, the VISTA framework provides organizations with a robust tool for holistic and actionable platform technology valuation. The illustrative examples demonstrate the framework’s applicability across diverse platform types and contexts, while the implementation guidance supports effective adoption and value capture.

The proposed Platform VISTA Framework is not a rigid, prescriptive model but rather a flexible tool designed to guide stakeholders across the pharmaceutical value chain. To effectively harness the potential of platform technologies, this framework should be approached as an iterative, collaborative process, with a strong emphasis on the integration of diverse perspectives. The valuation process should begin by assembling a cross-functional team, including experts in R&D, business development, finance, and regulatory affairs. This diversity ensures the framework benefits from a full spectrum of expertise, encompassing quantitative metrics and qualitative insights that collectively provide a holistic understanding of the platform’s value proposition. This multidimensional approach recognizes that platform valuation is not merely about numbers but also about interpreting near-term and long-term strategic, organizational, and market-level impacts.

A critical initial step in the valuation process is establishing an appropriate baseline for comparison, essential for quantifying a platform’s value relative to existing approaches. This baseline can be built by analyzing historical benchmarks from past internal projects—both platform-based and traditional—to assess improvements in key areas such as development speed and cost reduction. Where internal benchmarks are lacking, industry standards or data from comparable platform technologies can provide an external point of reference, which is especially valuable for early-stage assets that may have limited available data. For these early-stage platforms, qualitative insights can be gathered by examining analogous platforms within the industry, thus broadening the scope of the baseline and enhancing the depth of the initial assessment. By defining and applying key success metrics within the Platform VISTA Framework, stakeholders can better contextualize the platform’s potential and generate a more informed, accurate valuation. This structured baseline-setting approach, in conjunction with the initial assessment, provides a foundation to gauge progress and potential value creation as the platform advances through the development lifecycle.

The evaluation begins with an initial assessment, which seeks to provide an overview of the platform’s capabilities by applying each criterion within the framework. This phase involves gathering quantitative metrics, such as time savings, cost reductions, and efficiency gains, alongside qualitative assessments gathered through interviews with key stakeholders. For molecule-enabled platforms, emphasis should be placed on metrics such as adaptability and modularity in applying validated mechanisms of action across different therapeutic indications. Conversely, for design-enabled platforms, the initial assessment should focus on efficiency gains achieved through computational tools like AI-driven screening and high-throughput automation. Establishing this foundational understanding is crucial before moving on to more detailed analysis.

Following the high-level overview, stakeholders should conduct a “deep dive” evaluation phase that looks more closely at how each platform performs across different stages of drug development. This detailed analysis involves reviewing specific metrics such as early drug discovery efficiency, clinical development success rates, and manufacturing scalability. Here, stakeholders should incorporate both internal project data and industry benchmarks to assess how the platform stacks up relative to traditional methods and competing technologies. In the absence of sufficient quantitative data for early-stage platforms, case studies of similar technologies that successfully navigated regulatory or development milestones can provide valuable reference points, enhancing the understanding of expected timelines and risks.

To ensure a robust and balanced valuation, stakeholders should conduct a comparative benchmarking analysis that builds upon the baselining exercise by assessing the platform’s performance relative to both conventional drug development and competing platforms. While the baseline establishes foundational metrics by which the platform’s value can be tracked, comparative benchmarking goes further by positioning these metrics within broader industry standards and norms. Key metrics like “time to regulatory approval” and “clinical trial success rates” can be compared against industry averages, highlighting the platform’s competitive advantages in a larger context. Real-world examples of platform use cases—such as the reuse of validated safety data across indications—further add qualitative depth and demonstrate value beyond the baseline metrics alone. This benchmarking process allows stakeholders to assess how effectively the platform outperforms traditional drug development practices, providing clearer insights to guide decisions around further investment, strategic modifications, or partnerships.

Scoring these metrics requires a subjective yet structured approach tailored to the organization’s priorities. Metrics should be rated on a scale (e.g., 1 to 5 or 1 to 10) based on how well the platform meets each criterion. The importance of each metric can then be weighted to reflect the organization’s strategic focus. For example, a company that is interested in rapid market entry might prioritize “time to approval” and assign it a higher weight, while another focused on broadening their therapeutic pipeline might emphasize “adaptability” and “modularity.” This flexibility in scoring ensures that the evaluation aligns with the company’s unique objectives while also creating a quantifiable outcome that guides strategic decisions. It also encourages discussion and alignment among cross-functional teams, helping reconcile differing priorities, such as those of regulatory teams focused on safety and those of commercial teams focused on speed to market.

An important consideration when using the Platform VISTA Framework is the recognition of interdependencies between the various value drivers. For instance, the IP strength of a molecule-enabled platform, measured through its coverage across therapeutic areas, could influence its revenue diversification opportunities, enabling greater licensing potential or strategic partnerships. Similarly, the capability to attract top-tier talent, such as leading data scientists for a design-enabled platform, might enhance innovation outputs, reinforcing brand and market leadership over time. By understanding these interdependencies, stakeholders can anticipate how strengthening one area can yield compounded benefits across others, making for a more robust and cohesive strategy.

Risk assessment should also be integrated into the valuation process to understand potential barriers or uncertainties in the platform’s journey. Stakeholders can utilize a risk matrix to categorize potential risks—such as regulatory uncertainties, technical feasibility challenges, or market dynamics—rating each risk on factors like probability and potential impact. By understanding these risks and incorporating mitigation strategies into the overall valuation, stakeholders can develop a balanced view of both opportunities and potential hurdles.

Stakeholders should also conduct a scenario analysis to envision how the platform might perform under various strategic conditions, such as expanding to a new therapeutic area, scaling production to meet increased demand, or navigating shifts in regulatory landscapes. By modeling different scenarios, stakeholders can identify strategic inflection points, foresee challenges, and prepare targeted responses, thereby enhancing long-term planning and flexibility.

An iterative approach to applying this framework is essential, as platform assets are not static but evolve through various stages of development. As new data become available or the platform reaches new milestones, stakeholders should revisit the valuation to reflect these advancements. For instance, as a molecule-enabled platform achieves early regulatory success or a design-enabled platform optimizes its screening efficiency, those changes should be integrated back into the valuation to accurately represent the platform’s current state and adjust forward-looking projections. The framework, therefore, serves not only as a one-time assessment tool but also as an evolving guide, continually updated with insights to support agile, data-informed strategic decision-making.

Additionally, incorporating practical tools such as data analytics and collaborative software can support the evaluation process. Digital tools can assist in real-time data collection and analysis, making quantitative metrics more dynamic and enabling stakeholders to track changes effectively. Collaborative platforms facilitate real-time sharing of data, insights, and expert opinions across cross-functional teams, ensuring alignment throughout the evaluation. These tools help enhance engagement, simplify the scoring and weighting process, and foster a more interactive assessment.

While the Platform VISTA Framework offers a comprehensive approach to capturing the multifaceted value of platform technologies, it is not without limitations. One significant challenge is the inherent subjectivity in scoring and weighting criteria, which can vary widely between organizations based on differing strategic priorities, risk appetites, and stakeholder perspectives. The effectiveness of qualitative assessments, such as those related to talent attraction or brand influence, heavily depends on the experience and insight of individual evaluators, which may introduce biases or inconsistencies. Additionally, early-stage platforms often lack sufficient quantitative data, leading to a reliance on analogous case studies or expert judgments that may not fully represent the specific nuances of the asset being evaluated. Moreover, while the iterative nature of the framework ensures adaptability over time, it also requires sustained effort, regular data gathering, and stakeholder engagement, which can be resource intensive. Finally, the framework’s effectiveness is contingent upon the quality of available internal and external benchmarks, which may not always be readily accessible, especially for novel platform technologies that have limited market precedents. These factors collectively underline the importance of using the framework as a flexible guide rather than a definitive valuation tool, requiring continuous refinement and careful consideration of its inherent limitations.

#### Case study 1: Alnylam’s RNAi therapeutics platform.

Alnylam’s RNAi platform leverages Nobel Prize-winning science to silence disease-causing genes with unprecedented precision and potency. The platform’s core components include optimized siRNA molecules, advanced lipid nanoparticle delivery systems, and a modular conjugate approach for targeted tissue delivery (38). This enables Alnylam to rapidly develop therapeutics across diverse indications with a consistent mechanism of action.

The platform’s broad applicability is evident in Alnylam’s diversified pipeline spanning cardiometabolic, infectious, and central nervous system diseases. As of 2022, Alnylam had 12 clinical programs, with four approved products generating $800M+ in annual revenue (39). Alnylam’s partnership network includes top biopharma companies such as Sanofi, Regeneron, and Vir Biotechnology, with deal values exceeding $2B.

Applying the VISTA framework reveals Alnylam’s strong strategic value, with a RDI of 0.75 across four therapeutic areas and three major geographies, and a PSI of 38.4 reflecting 10+ high-impact alliances. The platform’s technical value is evidenced by a PTS 1.5× higher than industry benchmarks and a CTE of 1.4, with an average of 5 y from first-in-human studies to approval.

Alnylam’s adaptive value is demonstrated by its Innovation Velocity Index of $3B, with four new programs entering the clinic annually, and a TLR of 2.2, reflecting top-decile employee engagement and retention. With an EES of 4.5, Alnylam is seen as a partner of choice, with 90%+ alliance renewals and referrals.

The VISTA analysis underscores Alnylam’s position as the leading RNAi platform company, with a strong and sustainable value creation engine. However, it also highlights opportunities to further enhance value through geographic expansion and manufacturing efficiencies. Alnylam’s experience illustrates the power of the VISTA framework to holistically assess platform value and guide strategic optimization.

#### Case study 2: Genentech’s therapeutic antibody platform.

Genentech’s therapeutic antibody platform has been a cornerstone of the company’s innovation engine for over two decades. The platform integrates proprietary antibody discovery, optimization, and manufacturing capabilities to enable the rapid development of best-in-class therapies (40). This includes industry-leading antibody libraries, automated HTS, and flexible mammalian cell production systems.

Genentech has leveraged its platform to build a broad pipeline of over 30 clinical-stage programs across oncology, immunology, ophthalmology, and infectious diseases (41). The company’s track record includes multiple blockbuster antibody franchises such as Avastin, Herceptin, and Ocrevus, each generating $5B+ in annual revenue. Genentech’s platform has also been the basis for high-value partnerships with Adaptive Biotechnologies, Lodo Therapeutics, and others.

Through the VISTA framework lens, Genentech’s platform shows a RDI of 0.8, with significant sales from six therapeutic areas and global market presence. The platform’s PSI is 42.6, reflecting a diverse set of 15+ partnerships across codevelopment, in-licensing, and technology access deals worth over $5B in total value.

Genentech’s technical value is evidenced by a PTS 1.3× higher than industry averages, with 63% of preclinical candidates reaching Phase 3 compared to 48% for nonplatform antibodies. The platform’s CTE is 1.6, indicating a 60% faster development speed, with an average of 6.2 y from discovery to approval vs. 10+ y typical in biopharma. Genentech’s MER of 3.5 reflects its ability to produce antibodies at 70% lower COGS and 5× the scale of industry benchmarks.

The platform’s adaptive value is underscored by an Innovation Velocity Index of $4.5B, with three to five new clinical programs launched per year with an average peak sales potential of $1.5B. Genentech’s TLR of 2.5 reflects its world-class scientific team and entrepreneurial culture, while its EES of 4.8 captures the company’s strong repeat partnerships and technology outlicensing.

The VISTA assessment affirms Genentech’s therapeutic antibody platform as a key driver of its sustainable leadership in the biopharma industry. The analysis also points to potential areas for further value creation, such as expanding platform applications beyond oncology and increasing technology outlicensing. Genentech’s case illustrates the VISTA framework’s utility in identifying sources of competitive differentiation and strategic value for well-established platforms.

#### Case study 3: Moderna’s mRNA platform.

To further demonstrate the broad applicability of the VISTA framework, we analyzed Moderna’s mRNA platform, which has transformed the vaccine and therapeutic landscape. Moderna’s platform leverages proprietary mRNA chemistry, delivery systems, and manufacturing processes to enable the rapid development of vaccines and therapeutics across a wide range of diseases (42, 43). The COVID-19 pandemic provided a powerful proof-of-concept for the platform, with Moderna delivering a highly effective vaccine in record time.

Applying the VISTA framework, Moderna’s strategic value is evident in its RDI of 0.65, with programs in five therapeutic areas and global commercial reach for its COVID-19 vaccine. The company’s PSI of 35.8 reflects high-impact alliances with AstraZeneca, Merck, and Biomedical Advanced Research and Development Authority (BARDA) totaling over $3B. Moderna’s IPLS of 8.2 captures its dominant mRNA patent estate, with 150+ issued patents globally.

The platform’s technical value is demonstrated by a PTS 2× higher than average, with 70% of preclinical candidates advancing to human trials. Moderna’s CTE of 2.4 highlights an average development timeline of 4.2 y from discovery to approval, over 50% faster than industry norms. The company’s MER of 4.6 reflects its automated, digital manufacturing systems, producing mRNA at 10x the scale and 80% lower COGS than competitors.

Moderna’s adaptive value shines through its Innovation Velocity Index of $7.5B, with ten clinical programs advancing in parallel and an average peak sales potential of $1.5B. The company’s TLR of 2.3 reflects its entrepreneurial culture and 1800-person team of top scientists and engineers. With an EES of 4.2, Moderna is seen as a go-to mRNA partner, with 90%+ alliance renewals and referrals.

The VISTA analysis highlights Moderna’s mRNA platform as a paradigm shift in biopharma R&D, with the potential to accelerate the creation of transformative medicines. It also surfaces opportunities for Moderna to further enhance value through expanded therapeutic area applications and strategic technology outlicensing. Moderna’s case underscores the VISTA framework’s value in crystallizing the technical and strategic dimensions of novel platform technologies.

## Conclusion

Platform technologies represent a transformative shift in pharmaceutical R&D, offering scalable, modular approaches that challenge the traditional single-asset paradigm. By integrating insights from a systematic literature review (38 studies) and real-world case analyses (Alnylam, Genentech, Moderna), we developed the Platform VISTA Framework to quantify strategic, technical, and adaptive value domains. This framework highlights the potential to reuse safety data, expedite clinical recruitment, streamline manufacturing, and spur intangible organizational benefits—thus capturing dimensions often overlooked by traditional valuation methods.

Notably, our findings show how platform-based efficiencies can accelerate time-to-market and diversify portfolios in ways that reshape biopharma investment strategies. At the same time, qualitative gains—like enhanced brand equity, top talent recruitment, and IP fortification—underscore the broader competitive advantages conferred by robust platforms. While we demonstrate these outcomes through selected case studies, wider validation across other modality-specific platforms (e.g., gene editing, AI-driven design) remains a promising avenue for future research. By systematically codifying the many dimensions of platform value, the VISTA Framework can inform stakeholder decisions on R&D prioritization, portfolio optimization, and partnership strategies. Our hope is that this approach both clarifies the tangible and intangible payoffs of platform innovation and catalyzes further refinement of valuation methods for next-generation therapeutics.

## Supplementary Material

Appendix 01 (PDF)

## Data Availability

No new primary datasets were generated for this study. The systematic literature review sources (all 38 articles) are detailed in the main reference list and summarized in *SI Appendix*, Table S1 (5, 9, 44–79). All case study information was compiled from publicly available records and corporate reports (see references). Further inquiries can be directed to the corresponding authors.
