# Supplementary material for: Framework to identify innovative sources of value creation from platform technologies
Source: Proc Natl Acad Sci U S A. 2025 May 19;122(21):e2424665122. doi: 10.1073/pnas.2424665122 (PMC12130853; doi:10.1073/pnas.2424665122)
Supplement: Supplementary file 1 — Appendix 01 (PDF) [file pnas.2424665122.sapp.pdf]

Supplementary Information

Supplementary Table 1. Literature on Platform Technology Valuation in Biopharma

Ref	Citation	Platform Focus	Valuation Approach	Key Insights Supported
1	Gawer, A., & Cusumano, M. A. (2014). Industry platforms and ecosystem innovation. *J. Prod. Innov. Manag.*, **31**(3), 417-433.	Design-enabled	Qualitative	1, 5
2	Bode-Greuel, K. M., & Greuel, J. M. (2005). Determining the value of drug development candidates and technology platforms. *J. Commer. Biotechnol.*, **11**(2), 155-170.	Both	Quantitative	1, 2
3	Sherkow, J. S. (2016). Protecting products versus platforms. *Nat. Biotechnol.*, **34**(5), 462-465.	Both	Qualitative	2, 5
4	Frazier R, Khan N, Lunawat G, Rahul A (2020) Products and platforms: Is your technology operating model ready? Available at: https://www.mckinsey.com/capabilities/mckinsey-digital/our-insights/products-and-platforms-is-your-technology-operating-model-ready. Accessed March 2025.	Both	Qualitative	1, 3, 5
5	Pisano, G. (2006). Can science be a business. *Harv. Bus. Rev.*, **10**, 1-12.	Both	Qualitative	3, 5
6	Arora, A., & Gambardella, A. (1990). Complementarity and external linkages: The strategies of the large firms in biotechnology. *J. Ind. Econ.,* **38**(4), 361–379.	Both	Mixed	1, 3, 5
7	Booth, B. (2024). Biotech Risk Cycles: Assets and Platforms. Available at: https://lifescivc.com/2024/10/biotech-risk-cycles-assets-and-platforms/. Accessed March 2025.	Both	Mixed	2, 4
8	Scarlett, U. (2024). Navigating biotech investment cycles — the true north. *BioCentury*. Available at: https://www.biocentury.com/article/654643/navigating-biotech-investment-cycles-the-true-north-guest-commentary. Accessed March 2025.	Both	Mixed	2, 5
9	Jones, C. H., Madhavan, S., Natarajan, K., Corbo, M., True, J. M., & Dolsten, M. (2024). Rewriting the textbook for pharma: How to adapt and thrive in a digital, personalized and collaborative world. *Drug Discov. Today*, **29**(9), 104112.	Both	Mixed	3, 5
10	Leclerc, O., Suhendra, M., & The, L. (2022). What are the biotech investment themes that will shape the industry? *McKinsey & Company*. Available at: https://www.mckinsey.com/industries/life-sciences/our-insights/what-are-the-biotech-investment-themes-that-will-shape-the-industry. Accessed March 2025.	Both	Mixed	2, 5

Ref	Citation	Platform Focus	Valuation Approach	Key Insights Supported
11	Capra, E., Fougner, C., Leclerc, O., Mäkitie, A., Suberski, A., & Suhendra, M. (2023). What early-stage investing reveals about biotech innovation. *McKinsey & Company*. Available at: https://www.mckinsey.com/industries/life-sciences/our-insights/what-early-stage-investing-reveals-about-biotech-innovation. Accessed March 2025.	Both	Mixed	1, 2, 5
12	Bettanti, A., Lanati, A., & Missoni, A. (2022). Biopharmaceutical innovation ecosystems: A stakeholder model and the case of Lombardy. *J. Technol. Transf.*, **47**, 1948–1973.	Both	Mixed	3, 5
13	Vedantam, K. (2022). Everyone loves a platform, especially biotech investors. *Crunchbase News.* Available at: https://news.crunchbase.com/health-wellness-biotech/biotech-platform-investment/. Accessed March 2025.	Both	Qualitative	2, 4
14	Lubroth, P., & Colluru, V. (2022). TechBio KPIs: Is your discovery platform validated*? Medium*. Available at: https://pablolubroth.medium.com/techbio-kpis-is-your-discovery-platform-validated-c686f95105f7. Accessed March 2025.	Design-enabled	Mixed	1, 2
15	Zoldan, A. (2023). Biotechs are accelerating drug discovery by repurposing platforms: What this means for investors. *Nasdaq*. Available at: https://www.nasdaq.com/articles/biotechs-are-accelerating-drug-discovery-by-repurposing-platforms-what-this-means-for. Accessed March 2025.	Both	Mixed	2, 4
16	ZS Associates. (2024). Antibody–drug conjugates: Oncology's next revolution. *ZS Insights*. Available at: https://www.zs.com/insights/oncology-antibody-drug-conjugates-revolution. Accessed March 2025.	Molecule-enabled	Mixed	2, 5
17	ZS Associates. (2023). Why therapeutic platforms could provide a powerful innovation model for large pharma. Available at: https://www.zs.com/content/dam/images/custom/Why-therapeutic-platforms-could-provide-a-powerful-innovation-model-for-large-pharma-FINAL.pdf. Accessed March 2025.	Both	Quantitative	1, 2, 5
18	Bennett, K. (2024). Key strategies for effective technology evaluation. *BioProcess International*. Available at: https://www.bioprocessintl.com/bioprocess-insider/key-strategies-for-effective-technology-evaluation. Accessed March 2025.	Design-enabled	Qualitative	3

Ref	Citation	Platform Focus	Valuation Approach	Key Insights Supported
19	Torani, V., Noy, T., Patel, P., & Shah, S. (2024). Adopting a holistic approach to pharma/biotech asset evaluation. Available at: https://bluematterconsulting.com/insights/blog/holistic-approach-to-pharma-biotech-asset-evaluation/. Accessed March 2025.	Both	Mixed	1, 3, 5
20	David, F. S., & Belogolovsky, G. (2019). A strategic approach to pharma R&D portfolio planning. *Pharmaceutical Executive*. Available at: https://www.pharmexec.com/view/strategic-approach-pharma-rd-portfolio-planning. Accessed March 2025.	Both	Qualitative	2, 3
21	Deloitte. (2023). Building a biotech valuation – A Deloitte perspective. *Deloitte UK*. Available at: https://www.deloitte.com/uk/en/Industries/life-sciences-health-care/perspectives/building-a-biotech-valuation-a-deloitte-perspective.html. Accessed March 2025.	Both	Mixed	2, 4, 5
22	U.S. Food and Drug Administration. (2024). Platform Technology Designation Program for Drug Development (Draft Guidance for Industry). Available at: https://www.fda.gov/regulatory-information/search-fda-guidance-documents/platform-technology-designation-program-drug-development. Accessed March 2025.	Molecule-enabled	Qualitative	3, 4, 5
23	Chandra, A., & Mazumdar, S. (2024). Biotech asset valuation methods: A practitioner's guide. *J. Invest. Manag.*, **22**(1), 36–57.	Both	Quantitative	2, 4
24	Rottgen, R. (2018). Biotech valuation idiosyncrasies and best practices. *Toptal Finance Blog*. Available at: https://www.toptal.com/management-consultants/valuation/biotech-valuation. Accessed March 2025.	Both	Qualitative	2, 5
25	Bädeker, M., Schulze, U., & Greber, D. (2015). R&D Productivity 2014: A breakthrough year for biopharma. *Boston Consulting Group Report*. Available at: https://www.bcg.com/publications/2015/biopharmaceuticals-innovation-research-development-productivity-2014-breakthrough-year. Accessed March 2025.	Both	Qualitative	2, 5
26	EY (2023) How ecosystems can help fill the life sciences innovation gap. *Ernst & Young*. Available at: https://www.ey.com/content/dam/ey-unified-site/ey-com/en-us/insights/life-sciences/documents/ey-is-innovation-deficit-biopharma-v7.pdf. Accessed March 2025.	Both	Qualitative	3, 4, 5
27	Nasir, M., Bak, P., Gauldie, S., & Leo, C. (2022). From drug target inhibition to degradation: a TACtical strategy. *Biopharma Dealmak.*, B38-B40.	Molecule-enabled	Qualitative	2, 4

Ref	Citation	Platform Focus	Valuation Approach	Key Insights Supported
28	BayBridge Bio. (2022). Biotech platforms are out, products are back in. *BayBridge Bio Blog*. Available at: https://www.baybridgebio.com/blog/platform-underperform.html. Accessed March 2025.	Design-enabled	Quantitative	2, 4, 5
29	Paul, S. M., Mytelka, D. S., Dunwiddie, C. T., et al. (2010). How to improve R&D productivity: The pharmaceutical industry's grand challenge. *Nat. Rev. Drug Discov.*, **9**(3), 203–214.	Both	Quantitative	1, 2, 3
30	Hartmann, M., & Hassan, A. (2006). Application of real option analysis in pharmaceutical R&D project valuation. *R&D Manag.*, **36**(3), 343-354.	Both	Quantitative	2, 4
31	Tralau-Stewart, C. J., Wyatt, C. A., Kleyn, D. E., & Ayad, A. (2009). Drug discovery: New models for industry–academic partnerships. *Nat. Rev. Drug Discov.*, **14**(1-2), 95-101.	Both	Qualitative	3, 5
32	Munos, B. (2009). Lessons from 60 years of pharmaceutical innovation. *Nat. Rev. Drug Discov.*, **8**(12), 959–968.	Both	Quantitative	5
33	Scannell, J. W., Blanckley, A., Boldon, H., & Warrington, B. (2012). Diagnosing the decline in pharmaceutical R&D efficiency. *Nat. Rev. Drug Discov.*, **11**(3), 191–200.	Both	Qualitative	1, 3, 5
34	Melese, T., Lin, S. M., Chang, J. L., & Cohen, N. H. (2009). Open innovation networks between academia and industry: An imperative for breakthrough therapies. *Sci. Transl. Med.*, **15**(5), 502–507.	Both	Qualitative	3, 4, 5
35	Hodgson, J. (1995). Biotech's platform technologies lure big pharma. *Nat. Biotechnol.*, **13**(9), 945–947.	Both	Qualitative	1, 2, 5
36	Kessel, M. (2011). The problems with today's pharmaceutical business—an outsider's view. *Nat. Biotechnol.,* **29***(1), 27-33.*	Both	Qualitative	5
37	DiMasi, J. A., Grabowski, H. G., & Hansen, R. W. (2016). Innovation in the pharmaceutical industry: New estimates of R&D costs. *J. Health Econ.*, **47**, 20–33.	Both	Quantitative	5
38	Jones, A. (2007). Minimizing leakage of value from R&D alliances. *Nat. Rev. Drug Discov.*, **6**(9), 711–719.	Both	Qualitative	3, 5

Platform Focus: *Molecule-enabled* = platform built around a specific molecule modality or biologic technology; *Design-enabled* = platform built on a design or computational methodology; *Both* = general insights applicable to platform technologies broadly.
Valuation approach: nature of the framework or method discussed (qualitative conceptual analysis, quantitative modeling/data, or mixed).
Key insights: (1) multifaceted frameworks; (2) standard metrics; (3) structured cross-functional processes; (4) adaptive/ecosystem value; (5) broader context consideration.

Supplementary Table 2. The Platform VISTA Framework

Area of Value Creation	Molecule-Enabled Platforms	Design-Enabled Platforms	Key Metrics of Value Creation
**I. Drug Development Value Creation**			
**A. Early Drug Discovery**			
**Target & Lead Discovery**	• Leveraging validated mechanisms across indications	• AI-driven screening<br>• Predictive modeling<br>• HTS for faster candidate identification	• Lead identification time<br>• Candidate viability rate<br>• Cost efficiency<br>• Quality of lead (efficacy)
**Lead Optimization**	• Cross-therapeutic adaptability<br>• Modular design	• Enhanced HTS precision<br>• Computational predictions accuracy	• Development time reduction<br>• Optimization speed<br>• Resource utilization, efficacy improvement
**B. Clinical Development**			
**Clinical Trial Design**	• Reusable safety profiles<br>• Reliable biological mechanisms	• Optimized patient selection<br>• Data-driven trial design	• Clinical trial success rate<br>• Recruitment speed<br>• Cost reduction<br>• Patient retention rate
**Accelerated Development**	• Reduced need for repetitive validation in early phases	• Predictive models for safety and efficacy<br>• Faster IND-enabling data integration	• IND submission speed<br>• Regulatory interaction success<br>• Time to clinical trial initiation
**C. Manufacturing**			
**Process Efficiency**	• Standardized production processes adaptable to multiple products	• Automation in production, digitalized quality control<br>• Consistent protocols	• Cost reduction per batch<br>• Production timeline compression<br>• Process adaptability rate
**Scalability & Flexibility**	• Modular scalability across product lines, shared infrastructure	• Automated workflows<br>• Digitalized process control for seamless scaling	• Scale-up efficiency<br>• Batch consistency<br>• Cost savings at scale<br>• Response adaptability
**II. Commercial Value Creation**			
**A. Commercial Opportunities**			
**Revenue Generation**	• Expanded product portfolio from platform flexibility<br>• Out-licensing<br>• Strategic partnerships	• Service-based revenue through platform licensing<br>• Expanded market reach	• Revenue diversification index<br>• Partnership revenue<br>• Market penetration rate
**Market Entry**	• Speed to market with proven safety/effectiveness<br>• Cross-indication adaptability	• Platform-based expansion via licensing<br>• Improved market adoption rates due to established efficacy	• Market launch time<br>• Penetration speed<br>• Geographic and therapeutic expansion
	**B. Strategic Value**		
**IP Portfolio**	• Broad patent coverage across therapeutic areas<br>• Platform technology exclusivity	• Algorithm/process patents<br>• Data privacy and protection<br>• Geographic IP coverage	• IP strength index<br>• FTO<br>• Competitive positioning in IP
**Innovation Platform**	• Deepening biological and therapeutic expertise<br>• Diversification in therapeutic areas	• Advancements in computational tools<br>• Algorithmic optimization<br>• Platform evolution	• Innovation rate<br>• Technological advances<br>• Adaptability across therapeutic areas

III. Intangible Value Creation			
**A. Organizational Capabilities**			
**Talent Acquisition & Retention**	• Reputation as innovation leader attracts specialized expertise in biology, chemistry, and clinical research	• Computational and AI/ML talent growth<br>• Cutting-edge appeal draws top-tier data science professionals	• Talent retention rate<br>• Recruitment success<br>• Impact on innovation productivity
**Knowledge Expansion**	• Accumulated insights into biological processes<br>• Expanded clinical and manufacturing knowledge	• Refinement of algorithmic and process knowledge<br>• Data-sharing for cross-project learning	• Efficiency in knowledge sharing<br>• Cross-platform learning<br>• Knowledge transfer effectiveness
**B. Ecosystem Position**			
**Industry Leadership**	• Authority in pioneering therapeutic development<br>• Platform recognition in clinical reliability	• Recognized leadership in technological innovation and operational efficiency	• Industry partnerships<br>• Brand influence<br>• Partnership demand
**Platform Evolution**	• Expansion of validated biological mechanisms across new indications, therapeutic adaptability	• Advancement in computational models<br>• Predictive accuracy improvements<br>• Enhanced platform capabilities	• Platform enhancement rate<br>• Adaptability across new indications<br>• Appreciation in platform value

Abbreviations: AI – Artificial Intelligence; AI/ML – Artificial Intelligence/Machine Learning; FTO – Freedom to Operate; HTS – High-Throughput Screening; IND – Investigational New Drug; IP – Intellectual Property; MOA – Mechanism of Action; R&D – Research and Development
